# Identification of Genetic and Chemical Modulators of Zebrafish Mechanosensory Hair Cell Death

**DOI:** 10.1371/journal.pgen.1000020

**Published:** 2008-02-29

**Authors:** Kelly N. Owens, Felipe Santos, Brock Roberts, Tor Linbo, Allison B. Coffin, Anna J. Knisely, Julian A. Simon, Edwin W. Rubel, David W. Raible

**Affiliations:** 1 Department of Biological Structure, University of Washington, Seattle, Washington, United States of America; 2 Virginia Merrill Bloedel Hearing Research Center, University of Washington, Seattle, Washington, United States of America; 3 Department of Otolaryngology—Head and Neck Surgery, University of Washington, Seattle, Washington, United States of America; 4 Fred Hutchinson Cancer Research Center, Seattle, Washington, United States of America; 5 Department of Physiology and Biophysics, University of Washington, Seattle, Washington, United States of America; Stanford University School of Medicine, United States of America

## Abstract

Inner ear sensory hair cell death is observed in the majority of hearing and balance disorders, affecting the health of more than 600 million people worldwide. While normal aging is the single greatest contributor, exposure to environmental toxins and therapeutic drugs such as aminoglycoside antibiotics and antineoplastic agents are significant contributors. Genetic variation contributes markedly to differences in normal disease progression during aging and in susceptibility to ototoxic agents. Using the lateral line system of larval zebrafish, we developed an in vivo drug toxicity interaction screen to uncover genetic modulators of antibiotic-induced hair cell death and to identify compounds that confer protection. We have identified 5 mutations that modulate aminoglycoside susceptibility. Further characterization and identification of one protective mutant, *sentinel* (*snl*), revealed a novel conserved vertebrate gene. A similar screen identified a new class of drug-like small molecules, benzothiophene carboxamides, that prevent aminoglycoside-induced hair cell death in zebrafish and in mammals. Testing for interaction with the *sentinel* mutation suggests that the gene and compounds may operate in different pathways. The combination of chemical screening with traditional genetic approaches is a new strategy for identifying drugs and drug targets to attenuate hearing and balance disorders.

## Author Summary

Loss of sensory hair cells in the inner ear is observed in the majority of hearing and balance disorders, affecting the health of more than 600 million people worldwide. Exposure to environmental toxins and certain pharmaceutical drugs such as aminoglycoside antibiotics and some cancer chemotherapy agents account for many of these hearing and balance problems. Variation in the genetic makeup between individuals plays a major role in establishing differences in susceptibility to environmental agents that damage the inner ear. Using zebrafish larvae, we developed a screen to uncover genes leading to differences in antibiotic-induced death of hair cells and to identify compounds that protect hair cells from damage. The combination of chemical screening with traditional genetic approaches offers a new strategy for identifying drugs and drug targets to attenuate hearing and balance disorders.

## Introduction

Hearing loss and vestibular dysfunction are among the most common disorders requiring medical attention. Globally, over a third of older adults suffer from these conditions. Studies of both laboratory animals and humans reveal tremendous variation in hearing loss due to ageing as well as exogenous challenges such as ototoxic drugs and noise exposure, and show that this variability can be at least partially understood using genetic methods [1]–[5]. Rapid progress has been made using genetics to understand the molecular basis for congenital deafness [6], but adult-onset hearing loss is poorly understood despite its overwhelming prevalence. There are several examples where genes underlying familial adult-onset hearing loss have been identified [7]–[9], but these are rare diseases that account for a very small fraction of the enormous variation of acquired or age-related hearing and balance problems. Understanding how hair cell death is genetically modified by intrinsic and extrinsic challenges should lead to identification of new therapeutic targets for prevention of inner ear damage.

The initial cellular basis for most hearing loss and a significant proportion of balance problems is injury and loss of the mechanosensory hair cells that reside in the inner ear and transduce mechanical signals into electrical signals that are sent to the brain via the VIIIth cranial nerve. Treatments with aminoglycoside antibiotics or the cancer chemotherapeutics, cisplatin and carboplatin, often cause irreversible hearing loss [10]–[12] by killing hair cells. As with other forms of hearing loss, the effects of aminoglycoside exposure in humans and other outbred mammalian populations are widely variable and influenced by genetic factors [13]. For example, patients with mutations in mitochondrial genes, including mitochondrial 12S ribosomal RNA, show greatly enhanced sensitivity to aminoglycoside exposure [14]. However, these mutations also have variable penetrance, and are influenced by nuclear genes [15]. Mutations in mitochondrial rRNA are consistent with a model that aminoglycoside ototoxicity is the result of effects on mitochondrial translation similar to the antibiotic effects of prokaryotic translation inhibition [16].

Pharmacological approaches toward the prevention of hearing loss due to therapeutic drugs or chronic exposure to noise have centered primarily on antioxidants and cJUN kinase (JunK) inhibitors. While several studies support the idea that antioxidants or JunK inhibitors can limit aminoglycoside toxicity and cisplatin ototoxicity, the literature is complex and often the protection is dose dependent [11],[17]. Target based drug discovery is limited, however, by our understanding of the cellular pathways contributing to the inner ear pathology, and by the lack of methods to do broad screening of potential candidates.

The lateral line system of aquatic vertebrates is composed of mechanosensory organs on the surface of the head and body, and is used to detect variations in water pressure. Lateral line hair cells and their underlying support cells are organized into rosette-like clusters called neuromasts [18]. Zebrafish lateral line hair cells show structural, functional and molecular similarities to the mammalian inner ear hair cells (reviewed in [19],[20]). Like mammalian inner ear hair cells, the lateral line hair cells of zebrafish are killed by exposure to chemicals including aminoglycosides and cisplatin in a dose-dependent manner [21]–[25]. The accessibility of lateral line hair cells to visualization and manipulation, along with the cellular and molecular properties shared with inner ear hair cells, makes this system a good model for investigating genetic and pharmacological modulation of hair cell sensitivity to potentially ototoxic agents [26].

In this report, we describe a new approach for the identification of genes and pharmacological agents that modulate the sensitivity of hair cells to ototoxic agents such as aminoglycosides. We use this approach to identify 2 new pharmacological agents and 5 new mutations that protect against aminoglycoside-induced hair cell death. We describe a screen for small drug-like molecules that protect zebrafish lateral line hair cells and validate effectiveness of these newly discovered protective compounds in the mammalian inner ear. We report the initial results of an *in vivo* genetic screen for modulators of hair cell susceptibility to ototoxic drug exposure, including the identification of one such gene. These mutations provide an entry point for determining which molecular pathways can be modulated to alter drug response in the hair cells. Variation in these molecules may underlie differential susceptibility to drugs clinically and suggest likely points of regulation for prophylactic treatments in the future.

## Results

**Figure 1:**
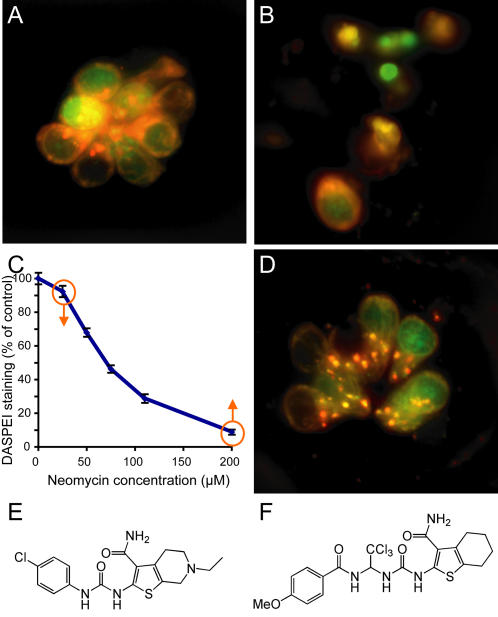
Screening for modifiers of aminoglycoside toxicity. (A) Neuromast from a control animal pretreated with 0.5% DMSO and stained with rapidly with FM 1-43FX (red) and the nuclear label Yo-Pro-1 (green). (B) Negative control pretreated with 0.05% DMSO for 1 hour followed by 200 µM neomycin treatment for 30 min. Hair cells are stained with FM 1-43FX (red) and Yo-Pro-1 (green). Hair cell loss, nuclear condensation and cytoplasmic shrinking are observed. (C) Dose-response function showing decreased hair cell labeling with DASPEI, a mitochondrial potentiometric dye, as a function of increasing neomycin concentration for wildtype zebrafish (N = 25–37 total fish per group, from triplicate experiments). Bars are SEM. Screens for increased or decreased susceptibility to hair cell loss were performed by treatment with either low, 25 µM, or high, 200 µM, neomycin doses, respectively, as highlighted by the orange arrows. (D) Neuromast pretreated with PROTO-2, a compound identified to provide protection against 200 µM neomycin exposure. (E,F) Show the structure for the identified compounds, PROTO-1 (E) and PROTO-2 (F), respectively.

Hair cells of the lateral line neuromasts in larval zebrafish form an easily identifiable rosette-like cluster that can be labeled with a variety of vital dyes and assessed in vivo (Figure 1A). The hair cells rapidly fragment and die upon treatment with 200 µM neomycin (Figure 1B). We have developed methods to systematically identify modulatory pathways altering hair cell response to aminoglycoside antibiotic exposure by taking advantage of in vivo labeling of lateral line hair cells with vital dyes. Figure 1C exemplifies this, showing that lateral line hair cells have a robust, highly reproducible response to different doses of aminoglycoside antibiotics [21],[23]. We reasoned that by examining animals treated with concentrations of neomycin at low or high ends of the dose-response curve, we should be able to identify modifiers that alter susceptibility to neomycin-induced hair cell death (Figure 1C).

### Small Molecule Screening for Protecting Compounds

**Figure 2:**
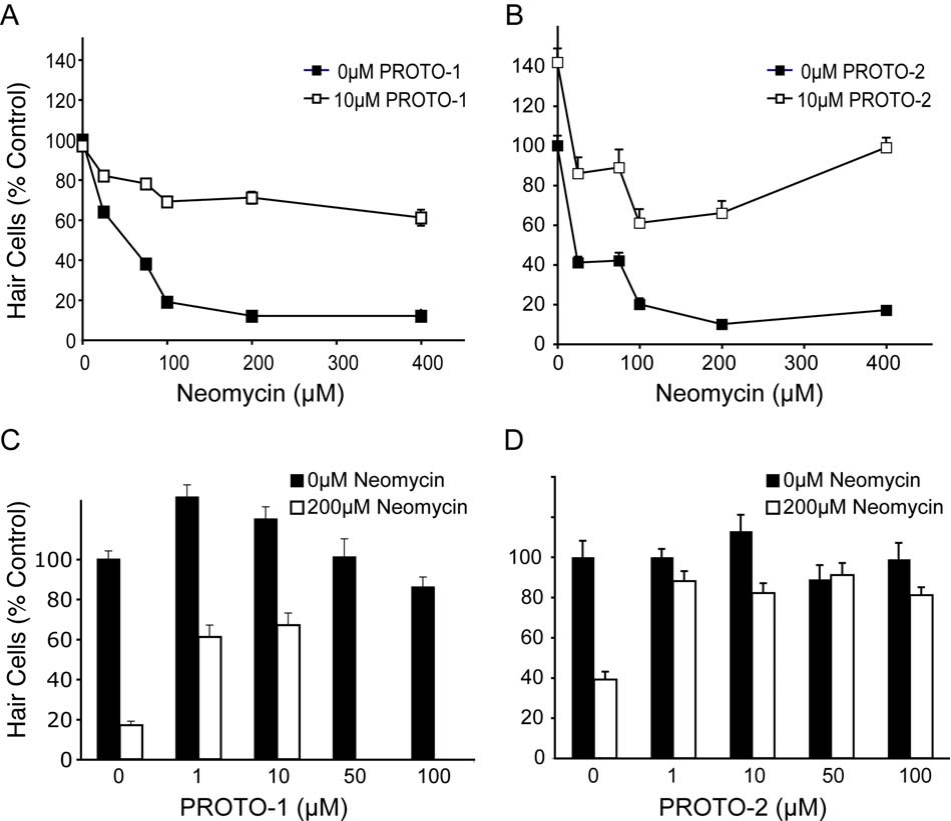
Ranges of protection for PROTO-1 and PROTO-2. Hair cells were vitally stained with FM1-43 and Yo-Pro-1, treated with PROTO-1 or PROT0-2 for 1 hour at various concentrations of compounds, then exposed to neomycin for 30 minutes, allowed 1 hr recovery in normal media. Graphs show mean hair cell counts for the SO1, SO2, OC1, and O1 neuromasts (+SEM) as percent of control (mock-treated, no neomycin exposure). Missing error bars indicate that was less than symbol size. (A,B) Neomycin dose-response curve showing effects of 10 µM PROTO-1 ((A), closed squares) and PROTO-2 ((B), closed squares) pretreatment in comparison to controls (without PROTO-1 or –2). (C,D) Profile of each compound at increasing doses without aminoglycoside and after 200 µM neomycin exposure. N = 10–20 fish per group.

To screen for small molecule modifiers, we pretreated 5 day post-fertilization (dpf) larvae with a chemically diverse library of 10,960 compounds before exposing them to 200 µM neomycin. Screening was initially carried out by labeling hair cells of 5 dpf larvae with a combination of a nuclear dye and a cytoplasmic dye (Yo-Pro-1 and FM 1-43, respectively), then pretreating larvae in 96-well tissue culture plates for 1 hour to a cocktail of five compounds and then exposing them to 200 µM neomycin. When protection was observed, the 5 potential contributors were evaluated singly to determine the active compound. Two compounds exhibited reliable and robust protection of hair cells from neomycin. An example of this protection is shown in Figure 1D, compared to treatment with neomycin alone (Figure 1B). Both compounds were benzothiophene carboxamides (Figure 1E and 1F), suggesting specific selection from the diverse library. We have named these compounds PROTO-1 and PROTO-2. We next compared the neomycin dose-response relationship in larvae pretreated with the compounds and controls (Figure 2). Figure 2A and 2B show that at a concentration of 10 µM both compounds show significant protection of hair cells over a broad range of neomycin concentrations, from 25 µM to 400 µM (p<0.0001 by two-factorial ANOVA). We also determined the dose-dependent effects of PROTO-1 and PROTO-2 to a fixed (200 µM) level of neomycin (Figure 2C and 2D). Pretreatment with 1 and 10 µM PROTO-1 resulted in significant protection of hair cells exposed to 200 µM neomycin compared to neomycin alone (p<0.0001, unpaired t-test). There was no significant difference in the protection provided by 1 and 10 µM PROTO-1 (p>0.10). Although exposure to 50 µM and 100 µM PROTO-1 alone did not alter viability, in combination with 200 µM neomycin these doses were lethal to larvae. Pretreatment with PROTO-2 provided significant protection of hair cells at all doses (p<0.0001, unpaired t-tests) with no dose-dependent difference (p>0.20). PROTO-2 was not lethal at any of the tested doses with or without neomycin.

Aminoglycosides are used clinically, despite their known ototoxicity, because of their broad spectrum of antibacterial actions. Compounds that could be used to limit their ototoxicity must not limit the intended therapeutic functions. We therefore had the University of Washington Clinical Microbiology Laboratory test the bacteriostatic and bactericidal activity of neomycin in the presence of PROTO-1 and PROTO-2. The minimum inhibitory concentration (3.25 µM) and minimum bactericidal concentration (6.5 µM) for E. *coli* ATCC 25922 was unchanged with or without 10 µM of either compound. This indicates that at least under standard in vitro assay conditions benzothiophene carboxamides do not inhibit aminoglycoside antibacterial activity.

### Screening for Genetic Modifiers

**Figure 3:**
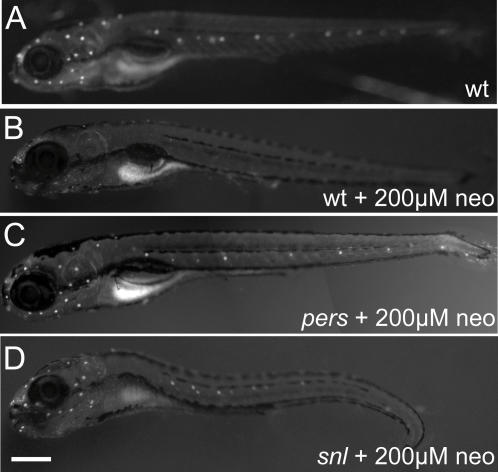
Mutations that confer protection against neomycin exposure. Larvae are labeled with DASPEI after 30 min exposure to 200 µM neomycin and 1 hr recovery in normal media. (A) Wildtype animal shows retention of hair cells in neuromasts after mock-treatment. (B) Wildtype animal shows loss of hair cells after aminoglycoside treatment. (C) *persephone* mutant animal shows robust protection of neuromasts against neomycin treatment. No morphological defects are observed. (D) *sentinel* mutant animal shows protection, along with sinusoidal body curvature. Bar = 200 µm.

To identify genetic modifiers of aminoglycoside-induced hair cell death, a standard F3 screening paradigm was used. Males were mutagenized with ethylnitrosourea following standard protocols [27], then crossed to wildtype females to produce F1 progeny. Mutagenesis was assessed by specific locus testing against unpigmented *mitfa* mutant animals [28], with a rate of about 1∶300. F2 families were produced from F1 individuals, and F3 larvae produced by pairwise intercrosses within each family. F3 larvae were treated at 5 dpf with either high (200 µM) or low (25 µM) concentrations of neomycin for 30 minutes to identify mutants that exhibit protection or heightened susceptibility of hair cells, respectively. Hair cells were then assessed with the vital dye DASPEI, which is differentially taken up by neuromast hair cells [29],[30]. Figure 3 shows untreated and neomycin-exposed wildtype animals, and two mutants with altered susceptibility. In contrast to the wildtype subject (Figure 3B), *persephone* mutants (Figure 3C) show robust staining indistinguishable from an untreated animal (Figure 3A). Animals homozygous for the *sentinel* mutation also retain robust staining; in addition they display a linked morphological phenotype, a variable sinusoidal morphology that begins to be apparent by 3 dpf (Figure 3D). While *persephone* mutants are homozygous viable, the *sentinel* mutation is lethal at approximately 10–12 dpf.

To date, we have identified 5 mutations that confer resistance and behave as simple recessive alleles. Complementation testing demonstrated that they affect different genes. We identified 5 additional mutations that confer resistance with more complex genetics, showing semi-dominant effects and/or interactions with modifying background loci. All mutations were transmitted to the next generation. We were surprised that all loci identified to date confer resistance, suggesting that affected genes normally act to promote cell death. The 5 simple recessive loci can be separated into two classes, mutations that have no apparent secondary phenotype (*persephone*, *trainman, bane*) and those with additional phenotypes (*sentinel, merovingian*). Animals homozygous for the *merovingian* mutation show reduced ear size and small otoliths (not shown).

**Figure 4:**
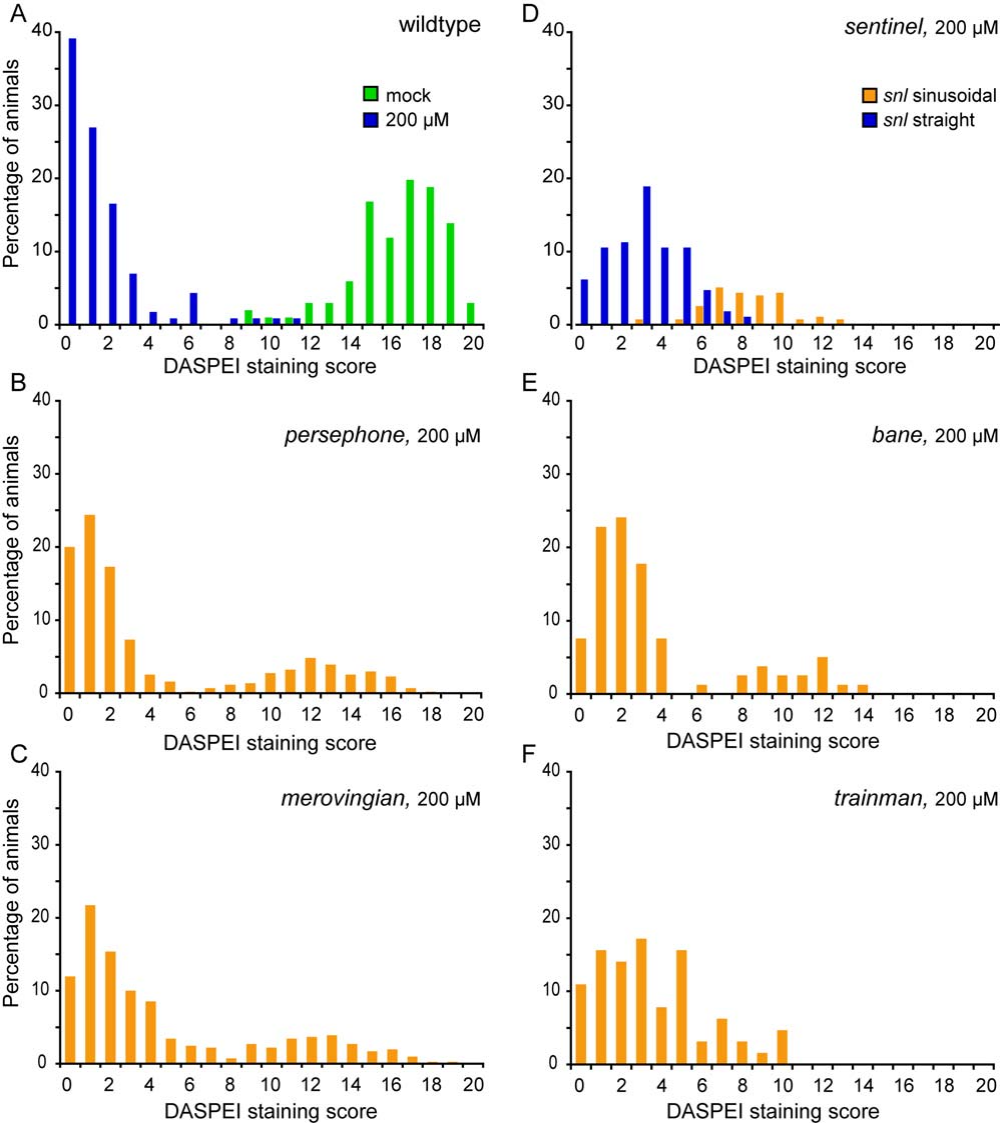
Hair cell retention after neomycin treatment in wildtype and mutant animals. Histograms show the fraction of animals with different levels of DASPEI staining. For each animal, 10 specific neuromasts are evaluated and assigned a score of 2 (normal staining), 1 (reduced staining), or 0 (no staining) for a maximum total score of 0–20. For each group, the distribution of animals given each DASPEI staining score is displayed as a percentage of the total number of animals to illustrate the phenotypic variation within the group; 40–80 animals were tested for each group. (A) Distribution of wildtype fish after mock treatment without neomycin (green bars) or after exposure to 200 µM neomycin (blue bars). (B–F) Distribution of progeny from crosses between heterozygous mutant carriers treated with 200 µM neomycin, showing both wildtype and mutant phenotypes. (B) *persephone*. (C) *merovingian.* (D) *sentinel*. Animals with sinusoidal bodies (later shown to be homozygous mutants) are represented by orange bars, and animals with wildtype body shape (wildtype or heterozygous siblings) are represented by blue bars. (E) *bane*. (F) *trainman*.

We have found that mutations differ dramatically in the relative resistance they confer against neomycin exposure (Figure 4). In wildtype animals, 200 µM neomycin exposure reliably reduces DASPEI staining to less than 10% of control untreated animals (Figure 4A). To examine the variability in phenotypes, we crossed heterozygous parents to produce offspring in typical Mendelian ratios (75% wildtype: 25% mutant progeny). Distributions show bimodality with robust DASPEI staining in 1/4 of the neomycin-treated progeny from crosses of heterozygous individuals for all 5 simple recessive mutations, as expected (Figure 4B–4F). Linked phenotypes cosegregate with resistance as shown for *snl* mutations (Figure 4D). Examples of mutations that confer near total resistance are shown in Figure 4B and 4C, and examples that confer only partial effects are shown in Figure 4D–4F. Partial protection may indicate that affected genes alter only one of several mechanisms involved in neomycin-induced cell death or that identified alleles may be hypomorphic and display only partial loss-of-function.

**Figure 5:**
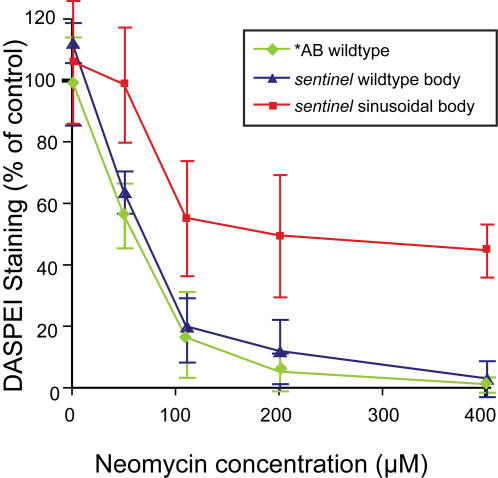
Dose dependent protection of *sentinel* mutants to neomycin. Hair cell loss as determined by DASPEI staining of progeny of *sentinel* heterozygous parents with wildtype body shape (blue) or sinusoidal body shape (red) are compared to wildtype *AB fish (green). Error bars are ±1 S.D. Mutants show robust, but partial, protection following 30 min neomycin exposure and one hour recovery.

The linked morphological features associated with *sentinel* mutants have allowed us to more fully characterize mutant phenotypes, since homozygous affected animals could be prospectively identified before directly testing the response to neomycin. We next tested whether *sentinel* mutants show altered response over the range of the aminoglycoside dose-response function (Figure 5). Animals were sorted by body phenotype as either wildtype or sinusoidal, and then exposed to different doses of neomycin. Animals homozygous for the *sentinel* mutation show robust, but partial, protection at all doses tested. Animals with wildtype body shape, including heterozygous mutants, are no different than the background *AB strain, demonstrating there are no effects of gene dosage. We also determined whether *sentinel* mutants show protection at later stages of development, since there are age-dependent differences in the dose-response to neomycin [22]. There is no change in the relative levels of protection by *sentinel* at 8–9 dpf (Figure S1), demonstrating that the mutation does not specifically confer protection by a general developmental delay.

### Molecular Identification of *sentinel* Mutation

**Figure 6:**
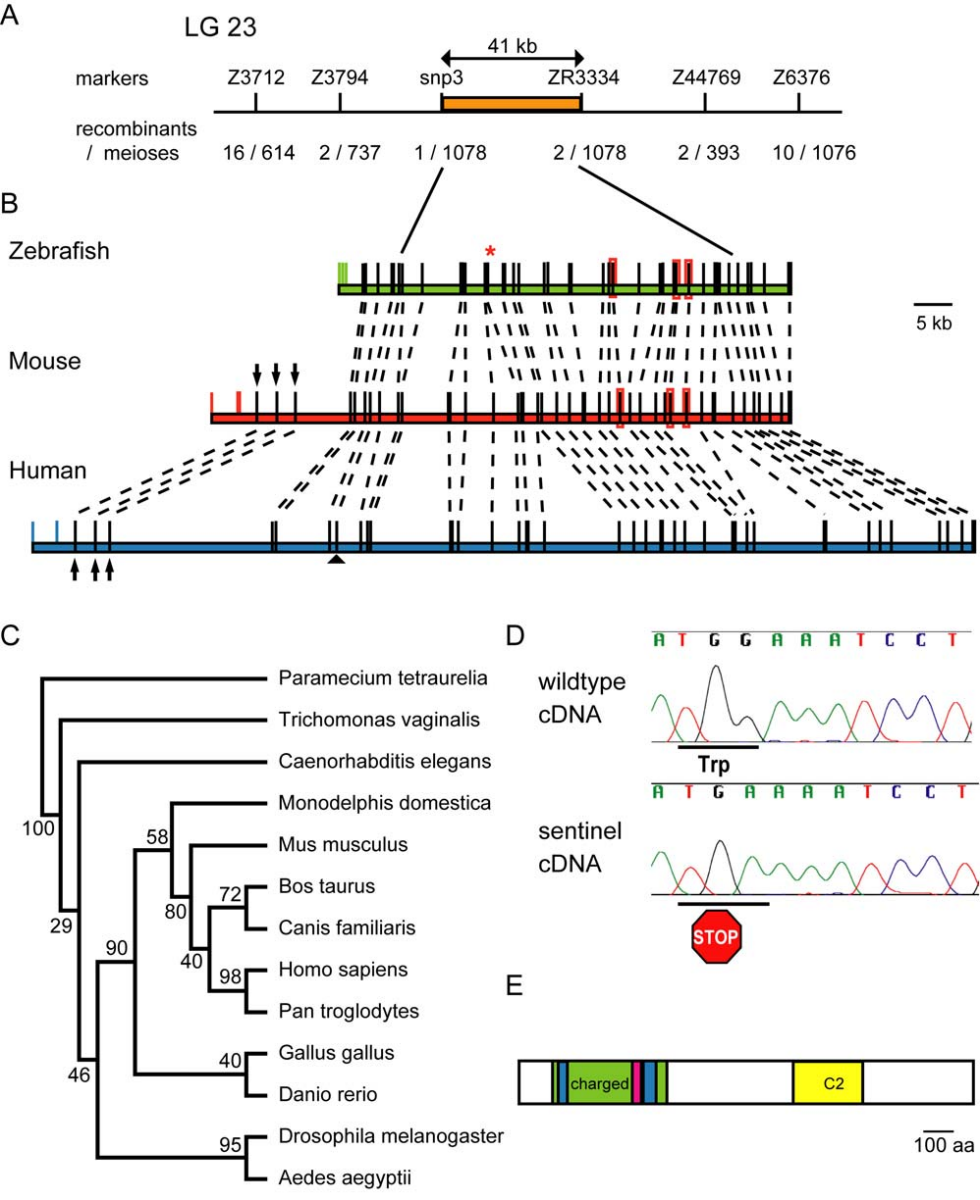
The *sentinel* mutation creates a stop codon in a novel vertebrate gene. (A) A schematic of chromosome 23 region illustrates fraction of recombinant chromosomes among informative meioses for genetic markers defining the *sentinel* linked region (orange box). (B) Colored bars represent the genomic structures of the *snl* orthologs from zebrafish (green), mouse (red), and human (blue). Black boxes denote exons, with dotted lines connecting orthologous regions between species, and colored bars represent introns. Divergent exons encoding 5′ UTR are shown as colored boxes. Three coding exons present only in the mammalian orthologs are noted with black arrows. Red rings highlight exons absent in human ortholog. The black arrowhead indicates the seven amino acids within exon 8 of zebrafish absent in the mammalian orthologs. A red asterisk marks the stop codon present in the *sentinel* allele within exon 14. (C), Phylogenetic tree of predicted proteins from *sentinel* orthologs. (D) cDNA sequence of wildtype zebrafish encoding tryptophan at amino acid 491 and of *sentinel* mutant bearing a stop codon. (E) Schematic of the predicted Sentinel protein including a C2 domain (yellow box) and a highly charged region (green box) with glutamine-rich basic clusters (blue boxes) flanking a lysine-rich acidic cluster (pink).

To determine the genetic location of the *sentinel* gene, we isolated 694 *snl* mutants and 234 *snl+* siblings based on the neomycin response phenotype of their hair cells. We detected cosegregation of the *sentinel* phenotype with markers [31] on chromosome 23 of zebrafish. Analysis of recombinant chromosomes revealed a 41 kb linked genomic region containing one candidate gene (Figure 6A) predicted to encode a 1541 aa protein with 38 exons. The predicted exon and intron boundaries are shown in Figure 6B. The boundaries of the linked region are positioned within the coding region (within introns 8 and 33) of this novel gene. Sequence of the coding regions and exon-intron junctions in *sentinel* cDNAs revealed a stop codon in exon 14 (Figure 6B, red asterisk, and Figure 6D) in place of a tryptophan. This alteration is predicted to truncate the protein at amino acid 491 with loss of 68% of the protein and is likely to lead to loss of function. The *sentinel* transcript is expressed ubiquitously in wildtype zebrafish (Figure S2). We observed attenuated expression in *sentinel* mutants, perhaps indicating that nonsense-mediated decay of the transcript occurs (Figure S2C).

Alignment of the zebrafish genomic region reveals homology to human (KIAA1345, 56% identity, 73% similarity) and mouse (RIKEN 5730509K17, 59% identity, 76% similarity) as well as to other vertebrates (Figure S3). The intron-exon structure between the zebrafish and mammalian orthologs is conserved with a few minor exceptions. We note looser homology to loci in Drosophila melanogaster, Aedes aegpytii, Caenorhabditis elegans, Trichomonas vaginalis and Paramecium tetraurelia genomes, suggesting that this is an ancient gene. The phylogenetic relationship between the predicted proteins is shown in Figure 6C. The Drosophila ortholog is annotated as two loci (CG18432 and CG18631) corresponding to the predicted N-terminal and C-terminal end of the zebrafish protein, indicating that they may encode a single transcript or be derived from a single ancestral locus. The predicted Sentinel protein contains a putative C2 domain [32] in the C-terminus (Figure 6E). The N-terminal third of the Sentinel protein is highly charged with two glutamine-rich acidic clusters flanking a lysine-rich basic cluster (Figure 6E). There is a notable absence of other recognizable domains.

### Genetic/Chemical Epistasis

**Figure 7:**
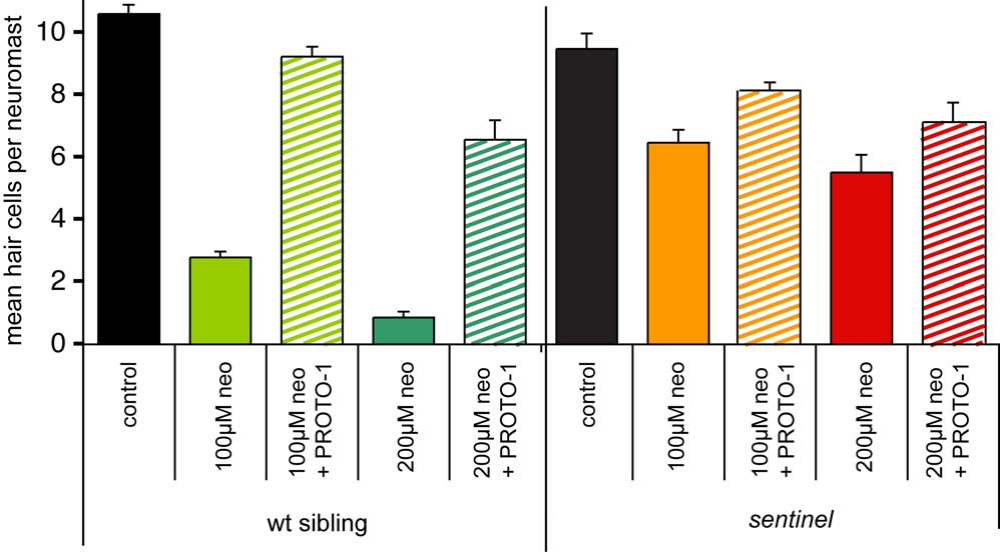
Epistasis analysis of *sentinel* and protective compounds. Neomycin dose-response relationship showing effects of 10 µM PROTO-1 against 100 µM or 200 µM neomycin exposure in wildtype and *sentinel* larvae. For each group, hair cells were pre-labeled with FM1-43FX. Animals were pretreated with PROTO-1 for 1 hour (or mock-treated), treated with neomycin and PROTO-1 for 1 hour, euthanized, and fixed. Hair cells of four neuromasts (left and right) were counted and the average number of hair cells per neuromast was determined. Number of hair cells in control animals (no PROTO-1, no neomycin) are shown with black bars, animals treated with only 100 µM or 200 µM neomycin are shown by solid colored bars, and animals treated with PROTO-1 and neomycin are shown by hatched colored bars. Error bars show 1 S.E.M. PROTO-1 and *sentinel* mutants show similar protection, and there is a small, statistically significant effect of the combined treatment of the mutation plus PROTO-1.

To begin elucidating possible molecular pathways regulating susceptibility, we tested for an interaction between *sentinel* mutants and PROTO-1. Both PROTO-1 treatment and *snl* loss of function result in substantial but incomplete protection against neomycin exposure. We tested whether exposure of PROTO-1 conferred any additional protection to *snl* mutants when exposed to 100 µM or 200 µM neomycin. Figure 7 provides these results for siblings (left) and *sentinel* mutants (right), comparing hair cell counts in control animals and fish exposed to neomycin with or without pretreatment for 1 hr in 10 µM PROTO-1. At both doses of neomycin, treatment with PROTO-1 provides a small amount of additional protection, over and above that provided by the *sentinel* mutation. Analyses by one-way ANOVA followed by pair-wise comparisons (Fisher's PLSD test) revealed that at both doses the additional protection provided by PROTO-1 was statistically reliable (p<0.01), but that even the combined effect did not provide complete protection (p<0.01).

### Determining Cellular Steps in Toxicity Altered by Modifiers

Attenuation of drug-induced hair cell death could result from a number of causes that are not directly linked to the activation of cell death or cell survival pathways. Some examples include the well-established link between mechanotransduction-dependent activity and aminoglycoside uptake and susceptibility [33]–[35], the relative resistance seen in young animals [23], and abnormalities of aminoglycoside uptake.

**Figure 8:**
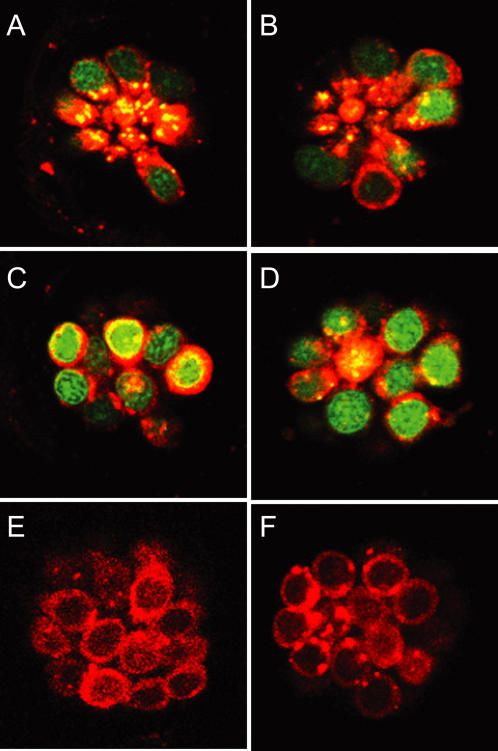
*sentinel* mutation does not affect transduction-dependent dye or aminoglycoside uptake. (A–D) Uptake of FM1-43FX after 45 sec exposure in wildtype (A,C) and *sentinel* mutants (B,D). Nuclei are labeled with Yo-Pro-1 (A-D). Confocal images of apical (A,B) and basal (C,D) optical sections through the hair cells. (E,F) Gentamicin-conjugated Texas Red uptake in wildtype (E) and *sentinel* mutant (F) animals after rapid 45 sec exposure.

Rapid uptake of the vital dye FM 1–43 is commonly used as an indicator of sensory hair cell mechanotransduction [36]–[38]. We compared the uptake of FM1-43FX in control (wild-type) fish, in *sentinel* mutants and in wild-type fish treated with PROTO-1 and PROTO-2 (Figure 8; Figure S4). Rapid entry of FM1-43FX into the hair cells of *sentinel* mutants (Figure 8B and 8D) is comparable to that of wildtype hair cells (Figure 8A and 8C). Similarly, PROTO-1 and PROTO-2 did not alter FM1-43FX uptake (Figure S4A, Figure S4B, Figure S4C), indicating that mechanotransduction-associated events appear intact with these modulators. In addition, examination of the neuromasts in *sentinel* mutants by light microscopy (compare Figure 8A and 8C to Figure 8B and 8D) reveals that hair cells are organized in the stereotypical rosette pattern found in wildtype animals. Together these results suggest that these modifiers do not act by blocking hair cell transduction or slowing development.

To test whether these modifiers alter drug entry, we evaluated whether fluorescently-tagged aminoglycosides [39] enter hair cells in the presence of modifiers. Both the aminoglycosides gentamicin (Figure 8E and 8F) and neomycin (not shown) tagged with Texas Red fluorophore enter *sentinel* hair cells with a rapid, 45-second, exposure. Similarly, PROTO-1 and PROTO-2 did not alter labeled gentamicin uptake (Figure S4D, Figure S4E, and Figure S4F). While these results do not rule out subtle changes in aminoglycoside uptake, they do show that there are no dramatic differences that might account for the broad range of protection seen. Hence, it appears most likely that modifiers affect steps in toxicity that occur after aminoglycoside entry.

**Figure 9:**
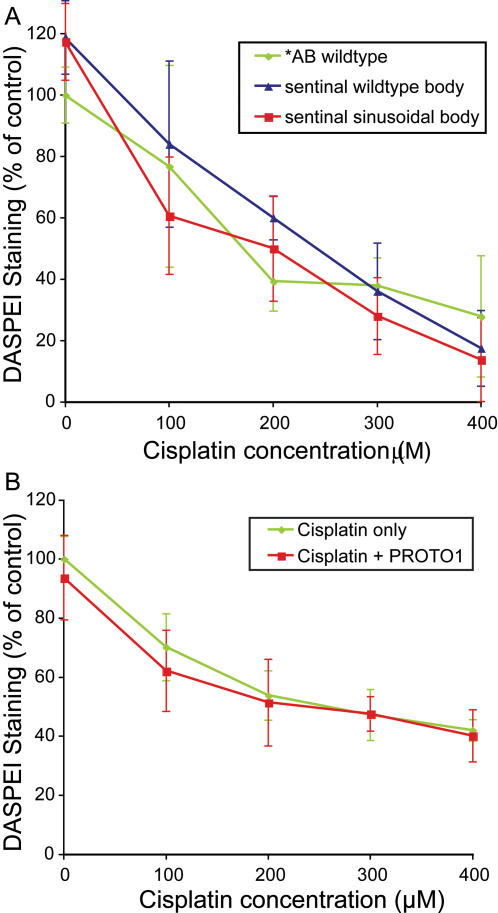
*sentinel* mutation and PROTO-1 do not protect against cisplatin. Hair cell survival was quantified using the vital dye DASPEI, and in each case DASPEI scores were normalized to those from wildtype, untreated fish. Fish (n≥12 fish per treatment group) were treated in cisplatin for 4 hours, then allowed to recover for 3 hours prior to DASPEI assessment. (A) Hair cell responses in wild-type versus *sentinel* mutants. No difference in the dose-response relationship was observed between wildtype fish (green), homozygous *sentinel* mutants (red, sinusoidal body), and *sentinel* siblings (blue, including heterozygous and homozygous wildtype sibling, straight body). (B) Response of cisplatin-treated hair cells from wildtype fish in the presence of the potentially protective compound PROTO-1. There is no difference between dose-response curves with (red) and without (green) PROTO-1. Error bars represent ±1 S.D.

Although the initial mechanism of hair cell death induced by aminoglycosides and cisplatin may be quite different, the later general cell death events are thought to be similar. To test whether these modulators alter cisplatin toxicity, we tested the effects of a range of cisplatin doses on *sentinel* mutants and on animals treated with PROTO-1. The response of sinusoidal *sentinel* mutants to cisplatin mirrored wildtype strains and siblings with wildtype body shape (Figure 9A). Thus, *sentinel* mutants are not protected against cisplatin-induced hair cell toxicity. Similarly, PROTO-1 did not protect against cisplatin-induced cell death (Figure 9B). The observation that *sentinel* mutants and fish exposed to PROTO-1 are relatively resistant to aminoglycoside-induced cell death but remain normally sensitive to cisplatin-induced cell death suggests that general cell death mechanisms are intact. We hypothesize that the *sentinel* mutation and PROTO-1 may abrogate aminoglycoside targets or early events in aminoglycoside-induced cell death that are not shared by cisplatin-induced cell death.

### Modifier Test in Adult Mammalian Utricles

**Figure 10:**
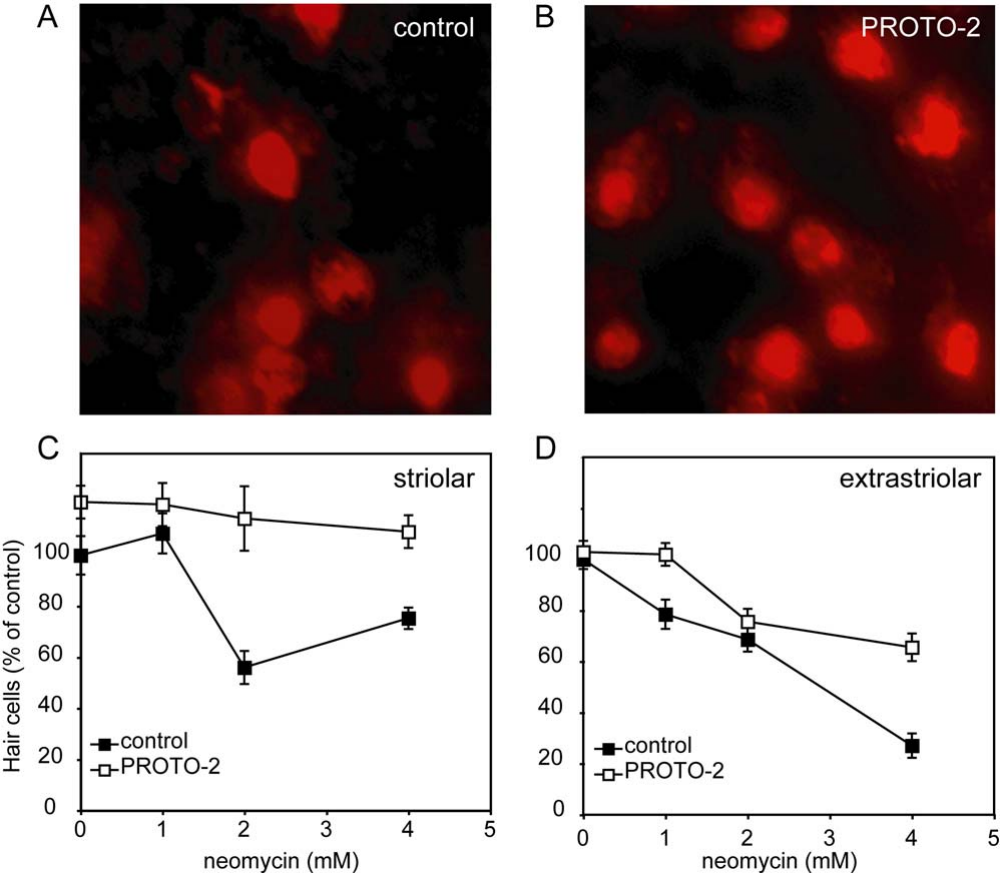
Protective compounds reduce neomycin toxicity in adult mouse utricle cultures. (A,B) Extrastriolar utricular hair cells stained with antibodies against calmodulin and calbindin after 4 mM neomycin exposure. An increased number of hair cells remain after PROTO-2 pretreatment (B) compared to control (A). (C,D) Neomycin dose-response curve showing effect of 10 µM PROTO-2 pretreatment on striolar (C) and extrastriolar (D) utricular hair cells. Counts were made at high magnification in areas of 900 µM^2^, converted to density, and averaged over the three sampled areas of each region for each utricle. Ten utricles were analyzed for each treatment group. Data were normalized relative to mock-treated controls (no PROTO drug, no neomycin).

Finally, we sought to determine whether modifiers we discovered in the zebrafish lateral line hair cell assay also confer protection to hair cells in the murine inner ear. While mutants for the mouse ortholog of *sentinel* are not yet available, a validated in vitro mammalian preparation of the mature mouse utricle has been used extensively to test protection of chemical modifiers [40]-[42]. We used the mouse utricle preparation to compare hair cell loss due to neomycin exposure between control utricles and utricles pretreated with PROTO-1 or PROTO-2. Figure 10 shows the neomycin dose-response relationship of striolar and extrastriolar hair cells in control utricles and utricles pretreated with PROTO-2. A two-factorial ANOVA (compound pretreatment×neomycin) showed significant protection using PROTO-2 (p<0.0001) in both the striolar and extrastriolar hair cell populations. PROTO-1 protection against neomycin was tested at 4 mM neomycin and showed significant striolar (p<0.0001), but not significant extrastriolar, protection. These results suggest that modifiers that can be rapidly identified and validated in the zebrafish lateral line system can have application in understanding ototoxicity in the mammalian inner ear.

## Discussion

Mechanosensory hair cells in the inner ear are susceptible to a wide variety of environmental insults. However, the large amount of variation in hearing and balance problems resulting from environmental or age-related challenges among normal individuals is neither well documented nor well understood. There is even large variance among individuals with the A1555G mutation in the mitochondrial 12S rRNA that increases susceptibility to neomycin toxicity [15]. We hypothesize that alterations in unidentified components of the network of cellular pathways involved in cell death and cell survival would confer resistance to ototoxic compounds. The absence of secondary phenotypes in some of our mutants supports the idea that variation affecting drug response can exist without other outward manifestation. Identification of the human orthologs of these genes may provide candidates involved in the variability underlying human hearing and balance disorders.

Our data suggest that hair cell death after neomycin treatment can involve multiple signaling pathways. Several mutations confer only partial protection against neomycin exposure. Although in some cases this might result from mutations that cause only partial loss of function, in the case of *sentinel* we suspect that the mutation is a functional null. The mutation introduces a stop codon early in the coding sequence and before the highly conserved regions. In addition, mRNA levels are reduced in *sentinel* mutants, suggesting nonsense-mediated decay. Together these observations suggest that gene function is completely lost, while protection against hair cell loss is only partial. Similarly, only partial protection is observed after treatment with maximal doses of PROTO-1 or PROTO-2. The idea that there are several possible responses to aminoglycosides is consistent with our previous observed variations in ultrastructural changes after aminoglycoside exposure [43].

The *sentinel* mutation also genetically distinguishes between aminoglycoside-induced and cisplatin-induced death; mutant animals are resistant to neomycin but still sensitive to cisplatin. Both aminoglycoside and cisplatin exposure have been proposed to result in oxidative stress [11],[44], raising the possibility that ototoxic compounds share similar mechanisms. If such shared mechanisms occur, the *sentinel* gene product must act upstream of these events. Treatment with PROTO-1 also offered no protection against cisplatin, suggesting that its cellular target acts specifically during aminoglycoside toxicity.

Inactivation of *sentinel* and treatment with PROTO-1 similarly alter the response of hair cells to neomycin treatment. Both modulators offer only partial protection against neomycin, offer no protection against cisplatin, and do not affect entry of FM1-43 or labeled aminoglycoside. Together these results suggest they work in common pathways. To test this idea, we performed epistasis experiments treating wildtype and mutant animals. While the effects of *sentinel* and PROTO-1 are not additive, there is a small but significant increase in protection when combined, suggesting that they may be accessing different cellular pathways to promote cell survival. Understanding similarities and differences among possible pathways will await the identification of the cellular targets of PROTO-1.

The identification of the *sentinel* gene highlights one strength of forward genetic screening, as it would be difficult or impossible to choose this gene *a priori* as a candidate regulator of mechanosensory hair cell death. No functional information is known about any of the *sentinel* orthologs. The only functional domain of note, the C2 domain, has been associated with calcium regulation and interaction with phospholipid membranes in signaling proteins such as protein kinase C or membrane trafficking proteins like Synaptotagmin [32]. However, the function of this domain has been demonstrated in only a few of the many proteins that contain it. Intriguingly, the D. melanogaster ortholog CG18631 was identified in a comparative bioinformatics screen as being associated with compartmentalized cilia-bearing organisms suggesting it may have a role in regulation of cilia [45]. Other members of this group include molecules related to intraflagellar transport (IFT) proteins and Bardet-Biedl syndrome (BBS)-related proteins associated with auditory function. In addition, the C. elegans K07G5.3 ortholog is enriched in ciliated neurons by SAGE analysis and localizes to ciliated sensory neurons [46]. Hair cells of the zebrafish lateral line and inner ear are also ciliated, bearing a microtubule-based kinocilium in addition to the actin-based stereocilia either throughout life (lateral line and vestibular epithelia) or during development (auditory epithelia/cochlea). However, the broad distribution of *sentinel* mRNA and lack of hair cell functional defects in mutants suggest that the gene product does not have a role specific to hair cells.

In addition to identifying possible therapeutic approaches, unbiased small molecule screening may reveal new molecular pathways that regulate hair cell death. This approach has been taken previously in a small molecule screen for compounds affecting zebrafish blood development; by identifying several compounds that affected prostaglandin metabolism, PGE2 was revealed as a regulator of haematopoiesis [47]. PROTO-1 and PROTO-2 are related benzothiophene carboxamides, suggesting that they may have the same molecular targets. Other benzothiophene carboxamides have previously been identified as HIV inhibitors, having effects on casein kinase, calcineurin and p53 [48]–[50]. Further work will be needed to determine whether any of these pathways modulate hair cell death.

Perhaps the most important contribution here is the suggestion that our screens can serve as templates for other research programs to identify other gene-drug interactions. Individuals respond remarkably differently to environmental exposures and drug treatment in most disease conditions. Efforts to understand population variation have centered on epidemiological and pharmacogenomic approaches [51]. However there are only a few cases in which the genes responsible for this phenotypic variability have been identified, such as for VKORC1-warfarin response or PON1-organophosphate toxicity [52],[53]. Genetic analysis may provide a systematic method to identify new molecules involved in cellular responses to drugs or disease.

## Materials and Methods

### Animals

Zebrafish embryos (*Danio rerio*) were produced by paired matings of adult fish in the University of Washington zebrafish facility by standard methods [54]. The *AB and WIK wildtype strains are maintained individually as inbred lines. Three to six-week-old CBA/CaJ mice were obtained from the Jackson Laboratory (Bar Harbor, ME) and maintained in the University of Washington Animal Care facility. All animal protocols were approved by the University of Washington Animal Care Committee.

### Vital Dye Staining

Larvae were transferred manually to baskets in 6-well culture plates containing defined E2 embryo medium. Baskets were constructed from the tops of 50 ml Falcon tubes in which the center of the lids were replaced with meshing. All treatment and wash volumes are 6 ml unless otherwise indicated. Hair cells of larvae were labeled with the following dyes: 1) FM 1-43FX (n-(3,3-ammoniumpropyl-dimethyl)ammoniumpropyl)-4-(4-(dibutylamino)styryl) pyridinium trichloride), an aminated derivative of FM1-43 (n-(3-triethylyammoniumpropyl)-4-(4-(dibutylamino)-styryl) pyridinium dibromide Invitrogen Molecular Probes, Eugene , OR) by immersing free swimming larvae in 3 µM FM 1-43FX in embryo medium for 30 or 45 s, followed by three successive rinses in embryo medium; 2) Yo-Pro-1 (Invitrogen Molecular Probes) at 3 µM for 1 hour followed by 3 rinses to selectively stain hair cell nuclei; or 3) DASPEI (0.005% final concentration, (2-{4-(dimethylamino)styryl}-N-ethylpyridinium iodide, Invitrogen Molecular Probes) in the final 15 minutes of the recovery period, and rinsed twice to brightly label mitochondria-rich hair cell cytoplasm. Larvae were anesthetized with MS222 (3-aminobenzoic acid ethyl ester, methansulfoneate salt, Sigma-Aldrich, St. Louis, Missouri) at a final concentration of 0.02% prior to imaging.

### Neomycin Treatment

Neomycin (Sigma-Aldrich, catalog no. N1142) was diluted in defined E2 embryo medium. Animals were treated with drug or embryo media (mock-treated controls) for times indicated, subsequently washed rapidly three times in fresh embryo medium and allowed to recover for one hour.

### Cisplatin Treatment

For cisplatin treatment, zebrafish larvae were exposed to 0–400 µM cisplatin (Sigma-Aldrich, catalog no. P4394) for 4 hours, rinsed several times in embryo medium and held 3 hours in the same media prior to DASPEI staining and visualization.

### Compound Screening

Larvae were stained with Yo-Pro-1 and FM 1–43 and then dispensed into 96-well glass bottom plates (Nunc, Rochester, New York) containing embryo medium (1–2 fish per well). Drug-like compounds from the Diverset E library (ChemBridge,San Diego, California), dissolved in 0.05% DMSO to a final concentration of 10 µM, were aliquoted into each well. Fish were incubated at 28.5°C for 1 hour. Neomycin was then introduced into each well at a final concentration of 200 µM and fish were incubated for an additional hour. Larvae were anesthetized with MS222 for immobilization. Visual assessment of hair cell integrity was performed in vivo using an inverted epifluorescent microscope. This allowed examination of the whole animal on the side of its body facing the objective and thus rapid evaluation of many neuromasts (∼20). In each row of the 96-well plate both positive (neomycin treated only) and negative (no neomycin) control animals were used for comparison to compound treatment. The entire plate of 96-well plate with 80 test wells and 16 positive or negative control wells was evaluated within one hour. Although intermediate responses were observed for some drugs, only those exhibiting robust protection were pursued for continued evaluation at this time.

To quantify changes in the hair cell response, hair cell survival was determined by counting the surviving hair cells from four neuromast, SO1, SO2, OC1 and O1 for 10–20 fish (i.e. 40–80 neuromasts). The percentage of surviving hair cells following treatment was calculated relative to mock-treated controls (no drugs or neomycin exposure).

### Minimum Inhibitory and Bactericidal Concentration Assays

Determination of the minimum inhibitory concentration (MIC) and the minimal bactericidal concentration (MBC) of neomycin alone and in the presence of 10 µM PROTO-1 or PROTO-2 were performed at the Clinical Laboratory of Microbiology at the University of Washington Medical Center as described by the National Clinical and Laboratory Standards Institute [55],[56].

### ENU Mutagenesis

Adult males from the *AB wildtype strain were mutagenized with 3 mM ethylnitrosourea (ENU) using standard procedures [27]). To assess the effectiveness of the mutagenesis, we performed a specific locus test of mutagenized males with homozygous *nacre* females; mutation of the *nacre* (*mitfa*) gene results in lack of pigment, which is readily apparent [28]. The ratio of progeny with a nacre-like pigment phenotype to total progeny was 1/300. Mutagenized males were then crossed to wildtype *AB females to produce F1 progeny. F2 families were derived from pairwise matings of F1 progeny of different mutagenized males.

### Genetic Screen

For each family screened, three to twelve F2 pairs were crossed and their progeny were examined for altered aminoglycoside response. Neomycin doses of 25 µM or 200 µM were used to screen for heightened susceptibility or protection, respectively. Ten neuromasts were evaluated on each fish for DASPEI staining and each neuromast was assigned a score of 0 for no/little staining, 1 for reduced staining, 2 for full staining [21], resulting in a final score of 0–20 for each fish. Scores were averaged and normalized to mock-treated controls. For initial analysis, 12–50 fish were assessed for typical and atypical responders (i.e. 120–500 neuromasts). Results were tabulated and chi-squared analysis was done to identify potential mutant strains of interest. Putative mutants were retested to confirm phenotype, outcrossed to *AB fish and tested again in the next generation to confirm transmission.

### Genetic Mapping

Heterozygous mutant carriers were outcrossed to the wildtype zebrafish from the polymorphic WIK strain for mapping. Hybrid *AB/WIK carriers of the hair cell modulator were then identified and crossed to produce progeny for marker analysis. At 5 dpf larvae were exposed to neomycin as described for the initial screen. To ensure accurate phenotyping, only individuals with the highest and lowest DASPEI staining scores after 200 µM neomycin treatment were retained as mutant and wildtype, respectively. For bulk segregant analysis, DNA was pooled from 20 wildtype or mutant individuals. Distribution of markers was compared to DNA from fin clips of *AB/WIK parents and founder grandparents. Microsatellite markers for each chromosome [31] were amplified by PCR and evaluated for cosegregation with mutant phenotypes. Linked markers were further evaluated with individual DNAs from 694 mutant fish and 234 wildtype fish (including both heterozygous and homozygous wildtype siblings). After determining initial linkage to chromosome 23, fine mapping identified Z3794 and Z44679 as flanking markers. A contiguous genomic sequence was then assembled using whole genome shotgun trace sequences produced by the Zebrafish Sequencing Group at the Sanger Institute (http://www.sanger.ac.uk/Projects/D_rerio/). Additional markers were developed to better define the linked region in *sentinel* mutants based on genomic sequence. *snp3* amplifies a single nucleotide polymorphism and *sat3334* is a sequence length polymorphism. They are amplified by the primers:

1. snp3_forward: GGGTGTCGAACTTGCACCTTTAAT
2. snp3_reverse: GTTGCTTAATTAGGCCTACAGCACT
3. sat3334_forward: CTTCATTCGCCCTCTGAACC
4. sat3334_reverse: GTGCACACTGTGATGTCGATAA

### cDNA Isolation and Sequencing/Molecular Biology

RNA was isolated from whole embryos at 62 hpf using Trizol according to manufacturer's specifications (Invitrogen, Carlsbad, California). Oligonucleotide primers were designed based on in silico genomic sequence. cDNA was synthesized using First Strand cDNA synthesis kit (Invitrogen) using oligo DT primers. The following primer pairs were used to amplify portion of cDNA spanning the recombination breakpoints:

1. pair 1 forward: AGGTTGAGGCTGGTTTGCCGA
2. pair 1 reverse: CTCTCAGTGCTTTCAGCTCCTTCCA
3. pair 2 forward: TTGTCAGACACACTCGACAGTTGCG
4. pair 2 reverse: TTGGGGTCGAGGCGAGATTCTG
5. pair 3 forward: AGATGGACGCCATCGCTTGCAT
6. pair 3 reverse: TCGTTCCAGCAGGGGTTTGGAC

Amplified products were cloned into pCR4 vectors using Topo TA cloning kit (Invitrogen). cDNA and genomic regions were sequenced from the vector T3 or T7 sites using Big Dye terminator v3.1 cycle sequencing chemistry (Applied Biosystems, Foster City, California).

### Comparative Genomics

Zebrafish cDNAs were aligned to known ESTs, cDNAs and genomic sequence from this region using Sequencher software (Gene Codes, Ann Arbor, Michigan). BLAST alignments of our cDNA sequences align with predicted cDNA (Genbank XM_693709/gi:125851476) amino acids 75-1040. Orthologs were identified from Genbank using BLAST and the corresponding predicted protein sequences were aligned with the *Danio rerio* predicted protein (XP_698801/gi:125851477): *Mus musculus* (NP_758478.1/gi:26986583), *Homo sapiens* (NP_001073991/gi:122937494), *Pan troglodytes* (XP_001159814 /gi:114593231), *Canis familiaris* (XP_536233/gi:73951827), *Bos taurus* (XP_595408/gi:119894226), Monodelphis domestica (XP_001369774/gi:126331991), *Gallus gallus* (XP_420777/gi:118090694), *Caenorhabditis elegans* (NP_492026/gi:U17508151), *Drosophila melanogaster* (NP_611229 and NP_611230/gi:24654454/28573534), *Aedes aegyptii* (EAT41051/gi:108876826), *Trichomonas vaginalis* (XP_001323414/gi:123480792), *Paramecium tetraurelia* (CAK70738/gi:124405296). ClustalW multiple sequence alignment software was used to align predicted proteins of orthologous genes using the Gonnet 250 matrix [57]. We used the Phylip 3.66 phylogeny software [58] to create a bootstrapped data set from the original alignment using Seqboot, then Protml to evaluate these datasets using the maximum likelihood method with a Jones-Taylor-Thorton model of amino acid substitution. A consensus tree was determined with Consense software by extended majority rule. Phylogenetic trees were draw with TreeView software [59]. Protein motif searching was performed using the Eukaryotic Linear Motif server (elm.eu.org).

### Gentamicin–Texas Red Conjugation

4.4 ml of gentamicin sulfate (Sigma-Aldrich, 50 mg/ml) and 0.6 ml succinimidyl esters of Texas Red (Molecular Probes, Eugene, Oregon; 2 mg/ml in dimethyl formamide) were agitated overnight to produce the conjugate solution [39]. The conjugated solution was diluted in embryo media to a final concentration of 200 µM gentamicin. Because neomycin contains six amino side groups, neomycin conjugation was performed similarly except that the ratio of neomycin to Texas Red was adjusted to 3:1 to ensure that on average one molecule of dye or less labeled each aminoglycoside molecule. To assess aminoglycoside entry, 5 dpf larvae were immersed in aminoglycoside-Texas Red conjugate for 45 seconds and rinsed in embryo medium four times before immediate imaging. Images were collected using Zeiss LSM5 Pascal confocal microscope. Z-stack images of neuromasts were collected.

### Utricle Preparation

Utricles were dissected and cultured in basal medium EAGLE supplemented with Earle's balanced salt solution and 5% fetal bovine serum following established procedures [40]. Neomycin sulfate stock solution (Sigma-Aldrich) prepared in sterile water was added directly to culture wells at the desired concentrations. The utricles were incubated for 4 hours in the compounds diluted with 0.05% DMSO or 0.05% DMSO alone for controls followed by a 24 hour incubation with neomycin.

### Utricle Immunohistochemistry

Utricles were fixed for 1 hour at 4°C in 4% paraformaldehyde in phosphate buffer. Following fixation, otoconia were removed by gently “washing” the surface with buffer through a 26 gauge syringe needle. Utricles were then incubated in blocking solution (2% bovine serum albumin, 0.4% normal goat serum, 0.4% normal horse serum and 0.4% Triton-X in PBS) for 3 hours at room temperature. Hair cells were double labeled in whole-mount preparations with a monoclonal antibody against calmodulin (Sigma-Aldrich) and polyclonal antibody against calbindin (Chemicon, Temecula, California) at 4°C diluted in blocking solution, 1∶250. The utricles were then rinsed and incubated for 2 hours at room temperature in secondary antibody diluted in blocking solution with biotinylated horse anti-mouse IgG (1∶200) and Alexa 594-conjugated goat anti-rabbit IgG. Utricles were mounted with Fluoromount-G (EMS, Hatfield, Pennsylvania) and coverslipped. The density of mouse utricular hair cells was determined by counting the number of hair cells in three randomly chosen nonstriolar regions and the number of striolar hair cells in three randomly chosen striolar regions from each utricle. Counts were made at high magnification in areas of 900 µM^2^, converted to density, and averaged over the three sampled areas of each region for each utricle. Ten utricles were analyzed in this way for each treatment group. Data were normalized relative to mock-treated controls (no PROTO drug, no neomycin).

## Supporting Information

Figure S1 Comparison of the dose response curve of 5 dpf versus 8–9 dpf sentinel mutants. Hair cell staining by DASPEI was assessed after neomycin exposure among progeny of *sentinel* heterozygous parents with wildtype body shape (blue) or sinusoidal body shape (red). The dose response curves of wildtype *AB control fish are shown (green). (A) Dose-response at 5 dpf. (B) Dose-response at 8–9 dpf. Error bars are {plus minus}1 S.D. Found at doi:10.1371/journal.pgen.0040041.sd001 (252 KB AI).
(0.26 MB AI)

Figure S2 Figure S2. In situ hybridization of biotinylated probes to the *sentinel* locus reveals ubiquitous expression. (A) Wildtype *AB larvae 64 hpf, antisense probe. (B) *AB larvae 64 hpf, sense probe. (C) *sentinel* mutants 64 hpf, antisense probe.
(1.69 MB AI)

Figure S3 Aligned sequence of Sentinel-related proteins. *Danio rerio* (XM_693709/gi:125851477), *Mus musculus* (NP_758478/gi:26986583), and *Homo sapiens* (NP_001073991/gi:122937494).
(0.03 MB DOC)

Figure S4 Protective compounds do not affect transduction-dependent dye or aminoglycoside uptake. (A-C) Rapid entry (45 sec) of 3 µM FM 1–43 (red) into untreated (A), PROTO-1 (B), or PROTO-2 (C) treated hair cells. Nuclei are labeled with Yo-Pro-1 (green). (D-F) Exposure to gentamicin-conjugated Texas Red results in rapid labeling of untreated (D), 10 µM PROTO-1 (E), or PROTO-2 (F) pretreated hair cells.
(5.40 MB AI)

## References

[1] Gates GA, Couropmitree NN, Myers RH (1999). Genetic associations in age-related hearing thresholds.. Arch Otolaryngol Head Neck Surg.

[2] Johnson KR, Zheng QY, Noben-Trauth K (2006). Strain background effects and genetic modifiers of hearing in mice.. Brain Res.

[3] Lanvers-Kaminsky C, Krefeld B, Dinnesen AG, Deuster D, Seifert E (2006). Continuous or repeated prolonged cisplatin infusions in children: a prospective study on ototoxicity, platinum concentrations, and standard serum parameters.. Pediatr Blood Cancer.

[4] Nelson EG, Hinojosa R (2006). Presbycusis: a human temporal bone study of individuals with downward sloping audiometric patterns of hearing loss and review of the literature.. Laryngoscope.

[5] Sill AM, Stick MJ, Prenger VL, Phillips SL, Boughman JA (1994). Genetic epidemiologic study of hearing loss in an adult population.. Am J Med Genet.

[6] Friedman TB, Griffith AJ (2003). Human nonsyndromic sensorineural deafness.. Annu Rev Genomics Hum Genet.

[7] Inoue H, Tanizawa Y, Wasson J, Behn P, Kalidas K (1998). A gene encoding a transmembrane protein is mutated in patients with diabetes mellitus and optic atrophy (Wolfram syndrome).. Nat Genet.

[8] Lynch ED, Lee MK, Morrow JE, Welcsh PL, Leon PE (1997). Nonsyndromic deafness DFNA1 associated with mutation of a human homolog of the Drosophila gene diaphanous.. Science.

[9] McGuirt WT, Prasad SD, Griffith AJ, Kunst HP, Green GE (1999). Mutations in COL11A2 cause non-syndromic hearing loss (DFNA13).. Nat Genet.

[10] Forge A, Schacht J (2000). Aminoglycoside antibiotics.. Audiol Neurootol.

[11] Rybak LP, Whitworth CA, Mukherjea D, Ramkumar V (2007). Mechanisms of cisplatin-induced ototoxicity and prevention.. Hear Res.

[12] Nakashima T, Teranishi M, Hibi T, Kobayashi M, Umemura M (2000). Vestibular and cochlear toxicity of aminoglycosides–a review.. Acta Otolaryngol.

[13] Versnel H, Agterberg MJ, de Groot JC, Smoorenburg GF, Klis SF (2007). Time course of cochlear electrophysiology and morphology after combined administration of kanamycin and furosemide.. Hear Res.

[14] Prezant TR, Agapian JV, Bohlman MC, Bu X, Oztas S (1993). Mitochondrial ribosomal RNA mutation associated with both antibiotic-induced and non-syndromic deafness.. Nat Genet.

[15] Guan MX, Fischel-Ghodsian N, Attardi G (1996). Biochemical evidence for nuclear gene involvement in phenotype of non-syndromic deafness associated with mitochondrial 12S rRNA mutation.. Hum Mol Genet.

[16] Guan MX, Fischel-Ghodsian N, Attardi G (2000). A biochemical basis for the inherited susceptibility to aminoglycoside ototoxicity.. Hum Mol Genet.

[17] Cheng AG, Cunningham LL, Rubel EW (2003). Hair cell death in the avian basilar papilla: characterization of the in vitro model and caspase activation.. J Assoc Res Otolaryngol.

[18] Dambly-Chaudiere C, Sapede D, Soubiran F, Decorde K, Gompel N (2003). The lateral line of zebrafish: a model system for the analysis of morphogenesis and neural development in vertebrates.. Biol Cell.

[19] Nicolson T (2005). The genetics of hearing and balance in zebrafish.. Annu Rev Genet.

[20] Whitfield TT (2002). Zebrafish as a model for hearing and deafness.. J Neurobiol.

[21] Harris JA, Cheng AG, Cunningham LL, MacDonald G, Raible DW (2003). Neomycin-induced hair cell death and rapid regeneration in the lateral line of zebrafish (Danio rerio).. J Assoc Res Otolaryngol.

[22] Murakami SL, Cunningham LL, Werner LA, Bauer E, Pujol R (2003). Developmental differences in susceptibility to neomycin-induced hair cell death in the lateral line neuromasts of zebrafish (Danio rerio).. Hear Res.

[23] Santos F, MacDonald G, Rubel EW, Raible DW (2006). Lateral line hair cell maturation is a determinant of aminoglycoside susceptibility in zebrafish (Danio rerio).. Hear Res.

[24] Williams JA, Holder N (2000). Cell turnover in neuromasts of zebrafish larvae.. Hear Res.

[25] Ou HC, Raible DW, Rubel EW (2007). Cisplatin-induced hair cell loss in zebrafish (Danio rerio) lateral line.. Hear Res.

[26] Ton C, Parng C (2005). The use of zebrafish for assessing ototoxic and otoprotective agents.. Hear Res.

[27] Solnica-Krezel L, Schier AF, Driever W (1994). Efficient recovery of ENU-induced mutations from the zebrafish germline.. Genetics.

[28] Lister JA, Robertson CP, Lepage T, Johnson SL, Raible DW (1999). nacre encodes a zebrafish microphthalmia-related protein that regulates neural-crest-derived pigment cell fate.. Development.

[29] Balak KJ, Corwin JT, Jones JE (1990). Regenerated hair cells can originate from supporting cell progeny: evidence from phototoxicity and laser ablation experiments in the lateral line system.. J Neurosci.

[30] Bereiter-Hahn J (1976). Dimethylaminostyrylmethylpyridiniumiodine (daspmi) as a fluorescent probe for mitochondria in situ.. Biochim Biophys Acta.

[31] Shimoda N, Knapik EW, Ziniti J, Sim C, Yamada E (1999). Zebrafish genetic map with 2000 microsatellite markers.. Genomics.

[32] Nalefski EA, Falke JJ (1996). The C2 domain calcium-binding motif: structural and functional diversity.. Protein Sci.

[33] Marcotti W, van Netten SM, Kros CJ (2005). The aminoglycoside antibiotic dihydrostreptomycin rapidly enters mouse outer hair cells through the mechano-electrical transducer channels.. J Physiol.

[34] Richardson GP, Forge A, Kros CJ, Fleming J, Brown SD (1997). Myosin VIIA is required for aminoglycoside accumulation in cochlear hair cells.. J Neurosci.

[35] Seiler C, Nicolson T (1999). Defective calmodulin-dependent rapid apical endocytosis in zebrafish sensory hair cell mutants.. J Neurobiol.

[36] Nishikawa S, Sasaki F (1996). Internalization of styryl dye FM1-43 in the hair cells of lateral line organs in Xenopus larvae.. J Histochem Cytochem.

[37] Gale JE, Marcotti W, Kennedy HJ, Kros CJ, Richardson GP (2001). FM1-43 dye behaves as a permeant blocker of the hair-cell mechanotransducer channel.. J Neurosci.

[38] Meyers JR, MacDonald RB, Duggan A, Lenzi D, Standaert DG (2003). Lighting up the senses: FM1-43 loading of sensory cells through nonselective ion channels.. J Neurosci.

[39] Steyger PS, Peters SL, Rehling J, Hordichok A, Dai CF (2003). Uptake of gentamicin by bullfrog saccular hair cells in vitro.. J Assoc Res Otolaryngol.

[40] Cunningham LL, Cheng AG, Rubel EW (2002). Caspase activation in hair cells of the mouse utricle exposed to neomycin.. J Neurosci.

[41] Cunningham LL, Matsui JI, Warchol ME, Rubel EW (2004). Overexpression of Bcl-2 prevents neomycin-induced hair cell death and caspase-9 activation in the adult mouse utricle in vitro.. J Neurobiol.

[42] Sugahara K, Rubel EW, Cunningham LL (2006). JNK signaling in neomycin-induced vestibular hair cell death.. Hear Res.

[43] Owens KN, Cunningham DE, Macdonald G, Rubel EW, Raible DW (2007). Ultrastructural analysis of aminoglycoside-induced hair cell death in the zebrafish lateral line reveals an early mitochondrial response.. J Comp Neurol.

[44] Wu WJ, Sha SH, Schacht J (2002). Recent advances in understanding aminoglycoside ototoxicity and its prevention.. Audiol Neurootol.

[45] Avidor-Reiss T, Maer AM, Koundakjian E, Polyanovsky A, Keil T (2004). Decoding cilia function: defining specialized genes required for compartmentalized cilia biogenesis.. Cell.

[46] Blacque OE, Perens EA, Boroevich KA, Inglis PN, Li C (2005). Functional genomics of the cilium, a sensory organelle.. Curr Biol.

[47] North TE, Goessling W, Walkley CR, Lengerke C, Kopani KR (2007). Prostaglandin E2 regulates vertebrate haematopoietic stem cell homeostasis.. Nature.

[48] Critchfield JW, Coligan JE, Folks TM, Butera ST (1997). Casein kinase II is a selective target of HIV-1 transcriptional inhibitors.. Proc Natl Acad Sci U S A.

[49] Gualberto A, Marquez G, Carballo M, Youngblood GL, Hunt SW (1998). p53 transactivation of the HIV-1 long terminal repeat is blocked by PD 144795, a calcineurin-inhibitor with anti-HIV properties.. J Biol Chem.

[50] Wang D, Westerheide SD, Hanson JL, Baldwin AS (2000). Tumor necrosis factor alpha-induced phosphorylation of RelA/p65 on Ser529 is controlled by casein kinase II.. J Biol Chem.

[51] Weinshilboum RM, Wang L (2006). Pharmacogenetics and pharmacogenomics: development, science, and translation.. Annu Rev Genomics Hum Genet.

[52] Humbert R, Adler DA, Disteche CM, Hassett C, Omiecinski CJ (1993). The molecular basis of the human serum paraoxonase activity polymorphism.. Nat Genet.

[53] Rieder MJ, Reiner AP, Gage BF, Nickerson DA, Eby CS (2005). Effect of VKORC1 haplotypes on transcriptional regulation and warfarin dose.. N Engl J Med.

[54] Westerfield M (2000). The Zebrafish book. A guide for the laboratory use of zebrafish (Danio rerio)..

[55] Wikler MA (2006). Methods for Dilution Antimicrobial Susceptibility Tests for Bacteria That Grow Aerobically..

[56] Wikler MA (2007). Performance Standards for Antimicrobial Susceptibility Testing..

[57] Chenna R, Sugawara H, Koike T, Lopez R, Gibson TJ (2003). Multiple sequence alignment with the Clustal series of programs.. Nucleic Acids Res.

[58] Felsenstein J (1989). PHLYIP–Phlylogeny Interference Package (version 3.2).. Cladistics.

[59] Page RD (1996). TreeView: an application to display phylogenetic trees on personal computers.. Comput Appl Biosci.

