# Supplementary material for: Identification of Genetic and Chemical Modulators of Zebrafish Mechanosensory Hair Cell Death
Source: PLoS Genet. 2008 Feb 29;4(2):e1000020. doi: 10.1371/journal.pgen.1000020 (PMC2265478; doi:10.1371/journal.pgen.1000020)
Supplement: Figure S3 — Aligned sequence of Sentinel-related proteins. Danio rerio (XM_693709/gi:125851477), Mus musculus (NP_758478/gi:26986583), and Homo sapiens (NP_001073991/gi:122937494). (0.03 MB DOC) [file pgen.1000020.s003.doc]

Owens Figure S3

CLUSTAL W (1.83) multiple sequence alignment

Homo_sapiens MNPREEKVKIITEEFIENDEDADMGRQNKNSKVRRQPRKKQPPTAVPKEMVSEKSHLGNP 60

Mus_musculus MNTNDEKMKIISEDFTGDGVDTEAGRRKKNSKVRRQQRKKKPPASPPEEMVSDKFQDGGQ 60

Danio_rerio ------------------------------------------------------------

Homo_sapiens QEPVQEEPKTRLLSMTVRRGPRSLPPIPSTSRTGFAEFSMRGRMREKLQAARSKAESALL 120

Mus_musculus QEVVEEEPETNLLSLTARRGPRSLPPIPSASRTGFAEFSMRERMREKLQAARSKAESALL 120

Danio_rerio -------------------------------------------MTEKLRAAKSKAASLLE 17

* ***:**:*** * *

Homo_sapiens QEIPTPRPRRLRSPSK-KELETEFGTEPGKEVERTQQEVDSQSYSRVKFHDSARKIKPKP 179

Mus_musculus RDVPTPRPRRLRSPSREKETETEFGTEPSTEVQDTQKEDDTKSYSRIKFRDSVRKIKSKP 180

Danio_rerio QESSIEPVSRLQSLQD-RNTVTRFDRDMDPDLRADE---DSSFAARLKFRDAARRAVKDK 73

:: . **:* . :: *.*. : . ::. : *:. :*:**:*:.*: .

Homo_sapiens QVPPGFPSAEEAYNFFTFNFDPEPEGSEEKPKARHRAGTNQEEEEG---------EEEEP 230

Mus_musculus QLPPGFPSAEEAYNFFTFNFDPEPEESEEKSPVKGGERAHHEDQEG---------EEG-T 230

Danio_rerio QVEAGLPTAEEAYNFFTFNFDPEPEQQRQKKKRRSRRREAEEEEEGDSEGEVEGEDEEGE 133

*: .*:*:***************** ..:* : .*::** :*

Homo_sapiens PAQGGGKEMDEEELLNGDDAEDFLLG-------LDHVADDFVAVRPADYESIHDRLQMER 283

Mus_musculus QAQERAKKTEEEELLNGKDAEDFLLG-------LDPTAHDFVAVRAAEYKSARIQLQKEK 283

Danio_rerio EVGELERQNEGEEESEARDEDDPLVGDEEDLFIIDQSAQDFLEVRRAEYVDYSRRLQRER 193

. :: : ** :. * :* *:* :* *.**: ** *:* . :** *:

Homo_sapiens EMLFIPSRQTVPTYKKLPENVQPRFLEDEGLYTGVRPEVARTNQNIMENRLLMQDPERRW 343

Mus_musculus EILFTPSRLTVPTYKKLPENIQPRFLEDEGLYIGARPEVARTNENIMENRLLIQEPGSKW 343

Danio_rerio DTLFVPSMRPVPASSKLAENTRPRFLEEEGLYVGERPHVCLTNLNILENRILKQAEGRKW 253

: ** ** .**: .**.** :*****:**** * **.*. ** **:***:* * :*

Homo_sapiens FGDDGRILALPNPIKPFPSRPPVLTQEQSIKAELETLYKKAVKYVHSSQHVIRSGDPPGN 403

Mus_musculus FGDDGRILALPSPIKPFPSRPSLTTREQSPKAGLETLYKKAEKYVHSRQHMIGSGDPPGN 403

Danio_rerio FGDDGRIVALPDPIKESSSRPPLFHLEDQLDPALQTVYRKALKSKHVNLYIAGMGDHQAD 313

*******:***.*** .***.: *:. .. *:*:*:** * * :: ** .:

Homo_sapiens FQLDIDISGLIFTHHPCFSREHVLAAKLAQLYDQYLARHQRNKAKFLTDKLQALRNAVQT 463

Mus_musculus FQLDIDISGLIFTHHPCFSREHVLASKLAQLYDQYLARQQRNKTKFLTDKLQALRKAVQT 463

Danio_rerio YQLDVDVSGLIFSHHPLFSREHVLGARLAQLYDQHLTRLHKNLTQLLTDKLNSLRNTIHN 373

:***:*:*****:*** *******.::*******:*:* ::* :::*****::**::::.

Homo_sapiens GLDPEKPHQSLDTIQKTINEYKSEIRQTRKFRDAEQEKDRTLLKTIIKVWKEMKSLREFQ 523

Mus_musculus SLNPEKPHQSLDTTQKTINEYKSEIRQTRKLRDAEQEKDRTLLKTIIKVWKEMKSLREFQ 523

Danio_rerio MLELHRGEALSQVSQQRIAEYKQEVRHTRQLRDVEQEKDRALLKNIIRLWKELKALRDFQ 433

*: .: . :. *: * ***.*:*:**::**.******:***.**::***:*:**:**

Homo_sapiens RFTNTPLKLVLRKEKADQKADEEAYEAEIQAEISELLEEHTEEYAQKMEEYRTSLQQWKA 583

Mus_musculus RFTNTPLKLVLRKEKVDPKLDEDAYEAEIQAEIHELLEEHMEEYATKMEEYRTSHQQWKA 583

Danio_rerio RFTNTPYKLFIRREKVERLQDEQEFESDIMAEVSELQAETEEEYQRKMNEYRRLHEEWKS 493

****** **.:*:**.: **: :*::* **: ** * *** **:*** ::**:

Homo_sapiens WRKVQRAKKKKRKQAAEEHPGDEIAEP-YPEEDLVKPSPPEPTDRAVIEQEVRERAAQSR 642

Mus_musculus WRKAQRAKKKKKKQTTEEHLEEEEAEESFPEEEVTKPIPPEPTDPAVIEQQVRERAAHSR 643

Danio_rerio WKRKQKALKKKQKKKRQEEEEEDEVSEEELGEEPEKPNPPEKPDTSILEEQVREKAARIR 553

*:: *:* ***:*: :*. :: .. *: ** *** .* :::*::***:**: *

Homo_sapiens RRPWEPTLVPELSLAGSVTPNDQCPRAEVSRREDVKKRSVYLKVLFNNKEVSRTVSRPLG 702

Mus_musculus RRPGEPTLIPELSLAGNVTPNDQCPRVEVSRREDVRRRSVYLKVVFNSKEVSRTVSRPLG 703

Danio_rerio RNPGEPVLIPELTVSGSITANEQCPRAEFARREDVAKRSLFVKVLYNDKEVSRTDSRTLN 613

*.* **.*:***:::*.:*.*:****.*.:***** :**:::**::*.****** **.*.

Homo_sapiens ADFRVHFGQIFNLQIVNWPESLTLQVYETVGHSSPTLLAEVFLPIPETTVVTGRAPTEEV 762

Mus_musculus ADFRVHFGQIFNLQIFNWPESLMLQVYETIGHSGTTLLAEVFLPIPETTLVTGRAPIEEV 763

Danio_rerio MDFRVHFGQIFNLKIVNWPESIKLQVFESVGSSS-TQLTEVCVPVPESSVLTGSAPSEEM 672

************:*.*****: ***:*::* *. * *:** :*:**::::** ** **:

Homo_sapiens EFSSNQHVTLDHEGVGSGVPFSFEADGSNQLTLMTSGKVSHSVAWAIGENGIPLIPPLSQ 822

Mus_musculus EFSSNQHVTLDHEGVGSGVPFSFEADGSNQLTLMTSGKVSHSVAWAVGENGIPLIPPLSQ 823

Danio_rerio EFSSNQRVTFNHEGVGSGVPFSFEADGTNPQTLLTSGKLSCCVSWAVGEDDVPLAPPSAQ 732

******:**::****************:* **:****:* .*:**:**:.:** ** :*

Homo_sapiens QNIGFRSALKKADAISSIGTSGLTDMKKLAKWAAESKLDPNDPNNAPLMQLISVATSGES 882

Mus_musculus QNIGFRSALRRADAISSIGTSGLTDMKKLAKWAAESKLDPNDPNHAPLMQLISVATSGES 883

Danio_rerio PGVAMHSGLRQMDAIACIGASGLNDMKKLGKWAAESRLDPNDPSNASIMQLLSVVSGGDM 792

.:.::*.*:: ***:.**:***.*****.******:******.:*.:***:**.:.*:

Homo_sapiens YVPDFFRLEQLQQEFNFVSDQELNRSKRFRLLHLRSQEVPEFRNYKQVPVYDREIMEKVF 942

Mus_musculus YVPDFFRLEQLQQEFNFVSEEELNRSKRFRLLHLRSQEVPEFRNYKQIPAYDREIMEKVF 943

Danio_rerio GVPEYFRLEQLQEEFNFLSEEELQRSRRYRLLRLRSQEVQEFRHFKCVPSTDREISEKVF 852

**::*******:****:*::**:**:*:***:****** ***::* :* **** ****

Homo_sapiens QDYEKRLRDRNVIETKEHIDTHRAIVAKYLQQVRESVINRFLIAKQYFLLADMIVEEEVP 1002

Mus_musculus QDYEKRLRDRNVIETKDHLDMHRATVAKYLQQVREAVVNRFLTAKHHFLLTDLVVEEEVP 1003

Danio_rerio QDYENRLKEGEIIDTKEHIDAHRALVAKYLQRVRESVINRFLIAKHHFILSDVISEDEVP 912

****:**:: ::*:**:*:* *** ******:***:*:**** **::*:*:*:: *:***

Homo_sapiens NIS-----ILGLSLFKLAEQKRPLRPRRKGRKKVTAQNLSDGDIKLLVNIVRAYDIPVRK 1057

Mus_musculus NISSEGSGILGLSLFKLAEQKRPLRPRRKGRKKVTAQNLSDGDIKLLVNIIRAYDIPVRK 1063

Danio_rerio SIGGPGAGVLGWNLFKLAEPKRPLKPRRKERKKVTAQNLSEGDIKLLVNIIRGYDIPVRR 972

.*. :** .****** ****:**** **********:*********:*.******:

Homo_sapiens PAVSKFQQPSRSSRMFSEKHAASPSTYSPTHNADYPLGQVLVRPFVEVSFQRTVCHTTTA 1117

Mus_musculus PVVSKFQQPSRSSRTFSEKQTASPSTHSPLHNADYPLGQVLVRPFVEVSFQRTICHTTTA 1123

Danio_rerio PYTGKAPVSAKSGRSFTETFTA-PASQTGQQGSEWPFAQPLIRPFVEVSFQRSVLQTSTA 1031

* ..* .::*.* *:*. :* *:: : :.:::*:.* *:**********:: :*:**

Homo_sapiens EGPNPSWNEELELPFRAPNGDYSTASLQSVKDVVFINIFDEVLHDVLEDDRERGSGIHTR 1177

Mus_musculus EGPNPSWNEELELPFRAPNGDYSTASLQSVKDDVYINIFDEVLYDILEDDRERGSGIHTR 1183

Danio_rerio EGPNPCWNEEIVLPFSAPNGDYSSTSLQSVRDEVFINVFDELLYDVVEDERERGNTIHTR 1091

*****.****: *** *******::*****:* *:**:***:*:*::**:****. ****

Homo_sapiens IERHWLGCVKMPFSTIYFQARFE------------------------------------- 1200

Mus_musculus IERHWLGCVKIPFSTIYFQARIDGTFKIDIPPVLLGYSKERNIIMERAFDSARSLSEGSY 1243

Danio_rerio IERHWLGSINIPFSTIYLQSRIDGTFKVCTPPVLLGYSKERSLGSEGGYDAVRSPSEGTF 1151

*******.:::******:*:*::

Homo_sapiens -----------------------------SQEDEKLLQATEKFQAECALKFPNRQCLTTV 1231

Mus_musculus ITLFITIEPQLVPGEPMREKMSDMLKKFDTQEDEKLLQATEKFQAECALKFPQRQCLTTV 1303

Danio_rerio LSLFITIEPQLVPGDTVREKSFSLLNSFDSQEVERVLLASEVFEKEASRRFSDRPCITTV 1211

:** *::* *:* *: *.: :*.:* *:***

Homo_sapiens IDISGKTVFITRYLKPLNPPQELLNVYPNNLQATAELVARYVSLIPFLPDTVSFGGICDL 1291

Mus_musculus TDMTGKTVFITRYLKPLNPPQELLHVYPNNPQATAELVARYVSLIPFLPDSVSFAGVCDL 1363

Danio_rerio IDINGKTVFVTRFIRPLNPPQELLDASPNGTQDSTELVARYVSLIPSLPDSVSFAGVCDL 1271

*:.*****:**:::*********.. **. * ::*********** ***:***.*:***

Homo_sapiens WSTSDQFLDLLAGDEEEHAVLLCNYFLSLGKKAWLLMGNAIPEGPTAYVLTWEQGRYLIW 1351

Mus_musculus WSTSDQFLDLLAGDEEEHAVLLCNYFLFLGKKAWLVMGSAIPEGPTAYVLTWEKNYYLIW 1423

Danio_rerio WSTCDQFLTLLAGDEEEHAVLLCNYFLSMGKRAWLIIGSAIPEGPTAYVLTYEQSRYVIW 1331

***.**** ****************** :**:***::*.************:*:. *:**

Homo_sapiens NPCSGHFYGQFDTFCPLKNVGCLIGPDNIWFNIQRYESPLRINFDVTRPKLWKSFFSRSL 1411

Mus_musculus NPCSGHCYGQFDAFCPLKSVGCLIGPDNIWFNIQHHDSPLRINFDVTKPKLWKSFFSRSL 1483

Danio_rerio NASTGQHYGQYDVFCPLQTIGCLINADNVWFNIQPYAAPVRMSFDISKPNLWKPFFSRAF 1391

*..:*: ***:*.****:.:****..**:***** : :*:*:.**:::*:***.****::

Homo_sapiens PYPGLSSVQPEELIYQRSDKAAAAELQDRIEKILKEKIMDWRPRHLTRWNRYCTSTLRHF 1471

Mus_musculus PYPGLSSVQPEELIYQHTDKAVAAELQDRIEKILKEKIMDWRPRHLTRWNRYCTSTLRHF 1543

Danio_rerio PDPGLSSVQPDALVYRRTDRTAAVELQDRIEKVLREKLMEWRPRHPTRWNRYCISTLRQF 1451

* ********: *:*:::*::.*.********:*:**:*:***** ******* ****:*

Homo_sapiens LPLLEKSQGEDVEDDHRAELLKQLGDYRFSGFPLHMPYSEVKPLIDAVYSTGVHNIDVPN 1531

Mus_musculus LPLLERSQGEDIEDDHRAELLKQLGDYRFSGFPLHMPYSEVKPLVEAVYSTGVHNIDLPN 1603

Danio_rerio LPKLELSGGREVAEEHRLELQSLLGEYRISGFPLHLPFSELRPIIEAVHSTGVHKVESPN 1511

** ** * *.:: ::** ** . **:**:******:*:**::*:::**:*****::: **

Homo_sapiens VEFALAVYIHPYPKNVLSVWIYVASLIRNR 1561

Mus_musculus VEFALAVYIHPYPKNVLSVWIYVASLVRNR 1633

Danio_rerio VEFALAVYVHPYPSNVLSVWVYIASLVSAY 1541

********:****.******:*:***:
